# Application direct anterior approach in pediatric femoral head and neck lesions

**DOI:** 10.1186/s13018-024-04721-z

**Published:** 2024-04-10

**Authors:** Jian Zheng, Yanting Zhang, Guoxin Nan

**Affiliations:** 1 Dongguan Children’s Hospital Affiliated to Guangdong Medical University, Dongguan Eighth People’s Hospital, Dongguan Institute of Pediatrics, Dongguan, 523326 China; 2 Chongqing Medical University, Chongqing, 400016 China; 3 Dongguan Eighth People’s Hospital, Dongguan, China; 4 Dongguan Key Laboratory of Orthopedic Biomaterials Research and Clinical Transformation, Dongguan, China; 5 Department of Pediatric Research Institute, Children’s Hospital of Chongqing Medical University, Chongqing, China

**Keywords:** Direct anterior approach, Femoral neck, Femoral head, Pediatric

## Abstract

**Background:**

Femoral neck is one of the high-risk areas for benign tumors and tumor-like lesions. Small range of lesions may also lead to pathological fracture, femoral head necrosis and other serious problems.

**Purpose:**

To investigate a new minimally invasive surgical approach to resect femoral head and neck lesions in children.

**Patients and methods:**

Retrospective study of 20 patients with femoral neck and femoral head lesions from February 2019 to March 2023 in our hospital. Among them, 14 were boys and 6 were girls, 17 were femoral neck lesions and 3 were femoral head lesions. The age of the patients ranged from 3.2 to 12.6 years, with a mean of 7.1 years. The patients were divided into group A and group B according to different surgical approaches; group A used the Smith-Peterson approach, Watson-Jones approach or surgical dislocation approach and group B used the DAA. Intra-operatively, incision length, operative time and blood loss were recorded in both groups. Group A consisted of 1 femoral head lesion and 8 femoral neck lesions, including 5 cases of bone cyst and 4 cases of eosinophilic granuloma. Group B consisted of 2 femoral head lesion and 9 femoral neck lesions. A total of 11 patients with different types of disease were included in group B, including bone cysts (3 cases), aneurysmal bone cysts (1 case), eosinophilic granulomas (6 cases), Kaposi's sarcoma (1 case).

**Results:**

The two groups of patients differed in terms of incision length (*P* < 0.05), operative blood loss (*P* < 0.05) and operative time (*P* < 0.05). At 6–48 months post-operatively, there were no significant differences in function and all patients had good hip function.

**Conclusion:**

The direct anterior approach is effective for resection of paediatric femoral head and neck lesions. It provides clear exposure of the surgical site, minimal trauma and does not compromise the integrity of the anterior musculature.

**Level of evidence**: III.

## Introduction

The femoral neck is one of the high-risk areas for benign tumors and tumor-like lesions. Due to its anatomical and biomechanical characteristics, even small-scale lesions can lead to pathological fractures, aseptic necrosis of the femoral head, and other serious problems [1]. Localized lesions commonly found in the femoral neck include eosinophilic granuloma, bone cysts, fibrous dysplasia, osteoid osteoma, herniation pits of the femoral neck, and chronic osteomyelitis [2]. For most of the benign lesions, conservative treatment can be adopted. However, for actively growing lesions that have the potential to erode adjacent bone and cause pathological fractures, surgical treatment is necessary [3].

For different lesions, there are different approaches, such as the Smith-Peterson approach and the Watson-Jones approach, and each approach has disadvantages and advantages [4]. If it is a lesion of the femoral head, the hip capsule should be opened, and the Smith-Peterson approach is preferred. This approach should expose a large incision and needs to cut the rectus femoris muscle, resulting in greater trauma, but the advantage is that the surgical field is widely exposed [5, 6]. If the lesion is in the femoral neck, the traditional approach is adopted by anterolateral Watson-Jones approach, but this approach is hard to expose the medial part of the femoral neck. If the lesion is in the medial part of the femoral neck, surgical hip dislocation is required, and the greater trochanter needs osteotomy. After the lesion is removed, the greater trochanter is reduced and fixed by screws. A secondary operation was required to remove the screw [7, 8]. Additionally, another known disadvantage of the Watson-Jones approach is the potential for superior gluteal nerve injury, abductor muscle injury, and subsequent Trendelenburg gait [9].

The direct anterior approach (DAA) was described by Carl Hunter in 1871 [10]. Thus reducing the risk of hip dislocation postoperatively [11]. The DAA does not require the severing of any muscles and tendons, preserves muscle and bone attachment, avoids tearing of muscle tissue, and still exposes the femoral neck and femoral head [12]. The DAA is similar to the Smith-Peterson approach in that it allows for a supine surgical position, which improves anesthesia and lung ventilation for the patient [13]. In recent years, with the popularity of minimal invasive technology concept, the DAA has been widely used in total hip arthroplasty (THA) [14]. However, there are not many applications in the field of pediatric orthopedics. The purpose of this study was to report our experience with the DAA in the removal of lesions in the femoral head and femoral neck in children and to evaluate the effectiveness of this approach.

## Methods

### Patients

**Fig. 1:**
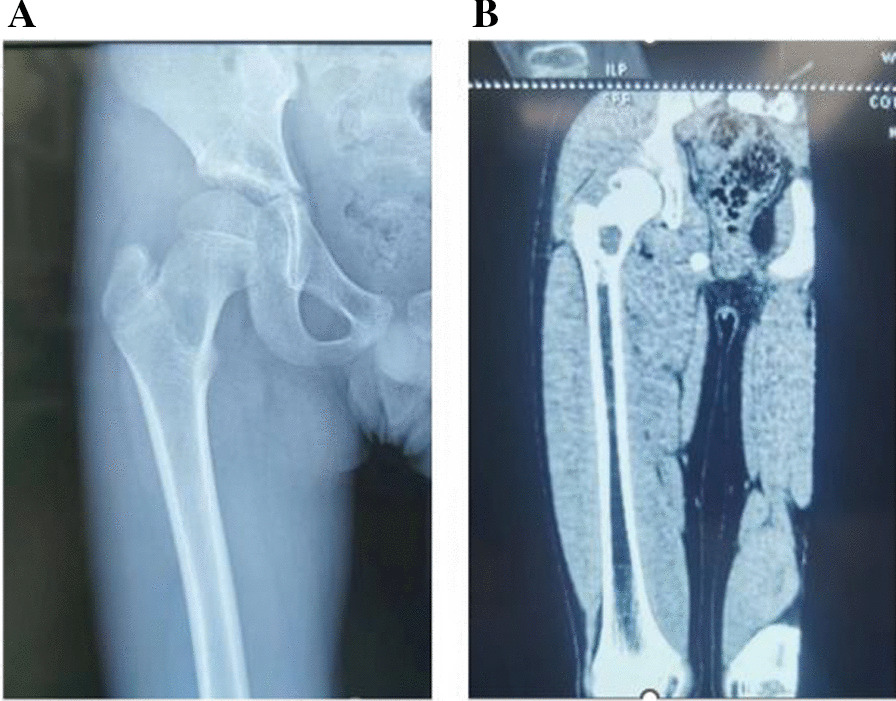
Imaging findings of femoral neck lesions: CT (**A**) and X-ray (**B**) findings of eosinophilic granuloma

A retrospective study of relevant treatments performed between March 2019 and March 2023 at our hospital. The inclusion criteria: (1) the age of onset was not more than 14 years old; (2) no pathological fracture; (3) The lesions were located between the base of the femoral neck and the head. Finally, 20 children met these criteria, and their data were screened for inclusion in this study. Among these children, there were 14 male children and 6 female children, femoral neck lesions appeared in 17 children, and femoral head lesions appeared in 3 children. The children ranged in age from 3.2 to 12.6 years, with an average age of 6.7 years. Before surgery, the patient was examined and diagnosed in detail using multiple imaging techniques such as X-ray, CT and MRI (Fig. 1). The surgical approach is divided into two groups, A and B. Group A includes the Smith-Peterson approach, the Watson-Jones approach, or the surgical hip dislocation approach. Group B includes direct anterior approach (DAA). A comparison was made between the two groups in terms of incision length, surgical time, blood loss, and post-operative functional differences. In order to ensure the rigour and reliability of the research, our research has been strictly approved by the Ethics Committee of our Institute.

### Surgical technique

All operations were performed by the same experienced surgeon. The surgeries were conducted under general anesthesia, and the children were positioned in a supine position. The DAA follows the neurovascular and muscular plane, especially the anatomical gap between the tensor fascia lata and the sartorius muscles [15]. It provides access to the hip joint by entering the superficial interval between the sartorius muscle (innervated by the femoral nerve) and the tensor fasciae latae (innervated by the superior gluteal nerve), and the deep interval between the rectus femoris (innervated by the femoral nerve) and gluteus medius (innervated by the superior gluteal nerve) [16]. However, the DAA does not require widening the interneural plane by removing the origin of the tensor fascia lata muscle on the iliac bone.

**Fig. 2:**
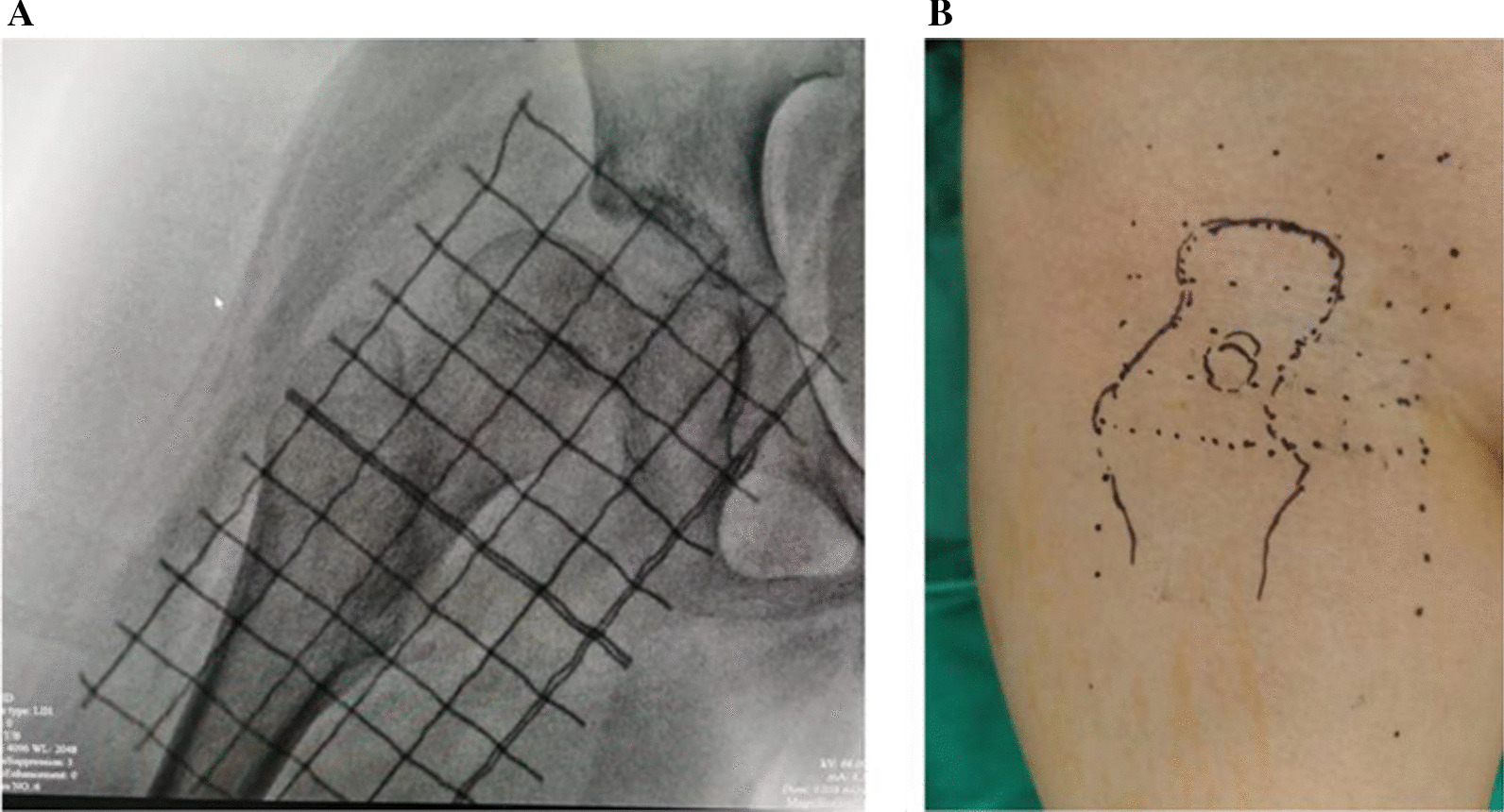
The grid localization method was used to locate the lesions: Grid positioning under X-ray (**A**) and body surface marking (**B**）

The grid localization method (as described in a previously published article [17]) was used to position and visualize the lesion through fluoroscopy with a C-arm, and the location of the lesion was described (Fig. 2). The surgical approach to DAA is described as follows. The skin and subcutaneous tissue were cut longitude-wise with the focal point as the centre. The incision length was about 3–4 cm, and then the sartorial muscle and the tensor fascia lata were bluntly separated to expose the interval. Entering along the interval reveals the deep muscles of the rectus femoris and gluteus medius. If it is necessary to further expose the femoral head, the hip joint capsule should be opened, the proximal end of the blunt free femoris rectus muscle should be lateral, and the proximal end of the femoris rectus muscle should be pulled inward to reveal the anterior part of the hip capsule, separate the fat pad in front of the joint capsule, and finally cut the joint capsule to expose the femoral head. If the femoral neck position needs to be exposed, opening the joint capsule is generally unnecessary. Patients treated through the surgical hip dislocation approach require intraoperative osteotomy at the greater trochanter. Following removal of the lesion, fixation with screws is necessary.

During the surgery, variables such as incision length, time from incision to lesion exposure, time for lesion removal, total operative time after lesion removal, and estimated blood loss until exposure completion were recorded. After the lesion was completely removed, thorough irrigation with normal saline was performed, followed by routine bone grafting. All pathological tissues were sent for diagnostic examination only. In order to prevent fracture, the hip was routinely immobilized with hip spica casting or orthotic devices for 6 weeks.

All cases in groups A and B were discharged from the hospital after recovery from surgery and all patients were followed up for a period of 6–48 months. All patients were assessed for hip function using the Harris Hip Score.

### Statistical analysis

All data are presented in box and violin plots and all data points and interquartile intervals are shown. The whiskers indicated the minimum and maximum values.Statistical significance was determined using unpaired Student’s t-test performed with Prism 8 software (GraphPad Software, San Diego, California, USA). A value of *P* < 0.05 was considered statistically significant. Harris Hip Score were normally distributed, and homogeneity of variance is expressed as mean ± standard deviation (SD).

## Results

**Fig. 3:**
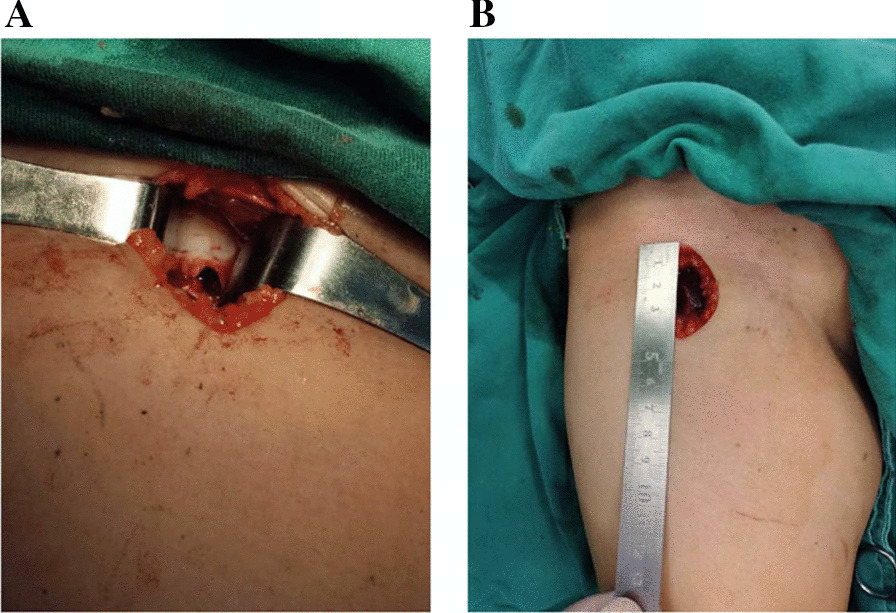
Exposure of the lesion through the DAA (**A**) and the length of the incision (**B**)

**Fig. 4:**
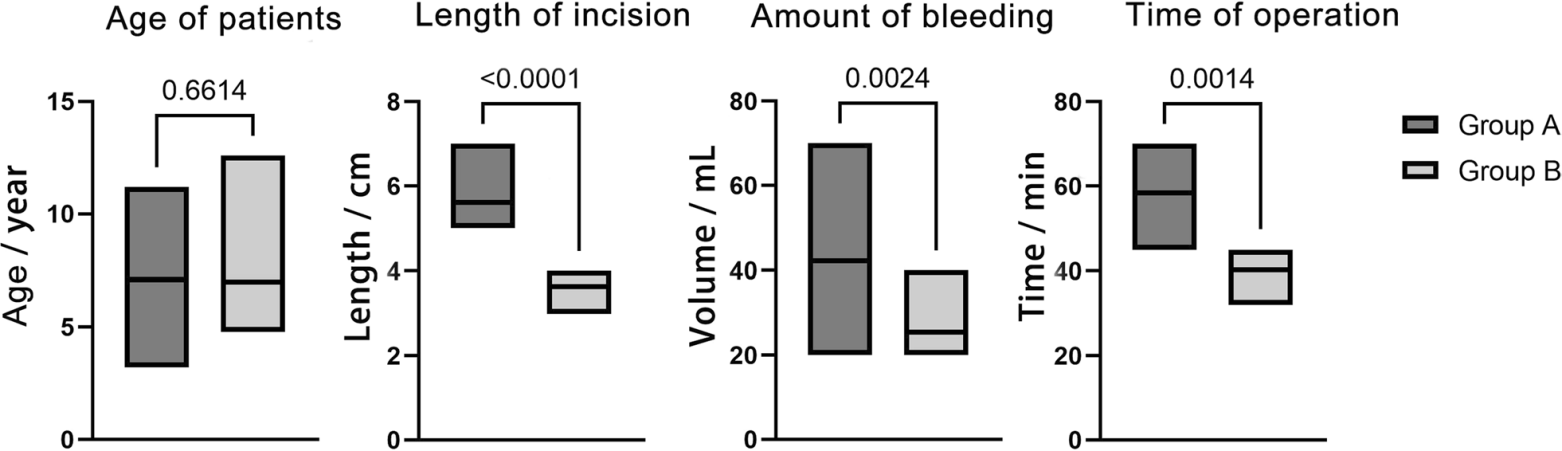
Comparison of age of patients, length of incision,amount of bleeding and time of operation between Group A and Group B

Group A had 9 cases, and Group B had 10 cases (Table 1). There was no significant difference in age between the two groups (*P* = 0.6614). In Group A, there was 1 case of femoral head lesion and 8 cases of femoral neck lesions, including 5 cases of bone cyst and 4 cases of eosinophilic granuloma. In Group B, there was 2 case of femoral head lesion and 8 cases of femoral neck lesions. In group B,10 patients with different types of disease, including bone cysts (3 patients), aneurysmal bone cyst (1 case), eosinophilic granuloma (6 patients) were included in this study. The aneurysmal bone cyst cases in Group B had excessive differences in haemorrhage compared to the whole and have been removed from the comparison of haemorrhage. It can be seen that patients with different types of diseases.There were differences between the two groups in terms of incision length (*P* < 0.05) (Fig. 3), surgical blood loss (*P* < 0.05), and surgical time (*P* < 0.05) (Fig. 4). Patients in both groups A and B were followed up for 6–48 months. Patients were evaluated for Harris hip function and compared 6 months after surgery. Following surgical debridement of the lesion, both group A (84.889 ± 3.689) and group B (84.5 ± 3.596) demonstrated good recovery of hip function despite utilizing different surgical approaches. However, the difference in functional scores between the two groups did not reach statistical significance (*P* > 0.05). There was no significant difference in the Harris Hip Scores of the children in groups A and B, which indicates that the children had good hip function, freedom of movement and normal gait (Table 2).

**Table 1:** Preoperative and postoperative case data

Group	Case	Age	Sex	Position of lesion	Approach	Length of incision (cm)	Time of operation (min)	Amount of bleeding (ml)	Type of lesion	Harris hip Score
A	1	3.2	F	Femoral head	S-P	6.0	55	20	Bone cysts	82
	2	6.5	M	Femoral neck	S-P	7.0	65	70	Eosinophilic granuloma	85
	3	7.8	M	Femoral neck	W-J	5.5	45	40	Bone cysts	88
	4	6.8	F	Femoral neck	W-J	5.0	55	45	Eosinophilic granuloma	87
	5	5.2	M	Femoral neck	W-J	5.5	63	50	Eosinophilic granuloma	80
	6	8.7	M	Femoral neck	S-D	5.0	70	35	Eosinophilic granulomas	84
	7	10.3	M	Femoral neck	S-D	5.5	65	30	Bone cysts	90
	8	11.2	F	Femoral neck	S-P	6.5	45	40	Bone cysts	87
	9	4.2	F	Femoral neck	S-P	5.0	62	50	Bone cysts	81
B	1	6.5	M	Femoral neck	DAA	3.5	38	20	Bone cysts	83
	2	5.9	M	Femoral neck	DAA	4.0	42	90	Aneurysmal bone cyst	81
	3	4.8	F	Femoral head	DAA	3.8	45	25	Eosinophilic granuloma	82
	4	7.2	M	Femoral neck	DAA	4.0	42	20	Eosinophilic granuloma	89
	5	6.6	M	Femoral neck	DAA	3.5	38	20	Bone cysts	86
	6	5.6	M	Femoral neck	DAA	3.0	40	25	Eosinophilic granuloma	85
	7	8.2	M	Femoral neck	DAA	3.8	45	20	Eosinophilic granuloma	89
	8	9.0	F	Femoral neck	DAA	3.5	38	30	Eosinophilic granuloma	87
	9	4.9	M	Femoral head	DAA	4.0	43	30	Bone cysts	81
	10	12.6	M	Femoral neck	DAA	3.5	32	25	Eosinophilic granuloma	91
*P* value		0.9796				< 0.05	< 0.05	< 0.05		

F, female; M, male; S-P, the Smith-Peterson approach; W-J, the Watson-Jones approach; S-D, the surgical hip dislocation approach

**Table 2:** Harris Hip Scores

	Time	Harris hip scores
Group A	6 month	84.889 ± 3.689
Group B	6 month	84.5 ± 3.596

## Discussion

The femoral head is located in the hip joint, and the hip capsule must be opened intraoperatively to remove the femoral head lesion [13]. For most pediatric surgeons, they usually choose the Simith-Peterson approach, which allows clear exposure of the surgical area [18], but the approach involves a larger incision and often requires severing the rectus femoris muscle, which takes a longer time to suture. In addition, due to postoperative damage to the rectus femoris muscle, the patient needs to wait for the muscle to fully heal before appropriate joint activity can be initiated. If a femoral neck fracture or lesion is encountered, the Watson-Jones approach is usually used. This approach, which is entered from the lateral side of the hip, can easily treat the basal lesions of the femoral neck with relatively good results [8]. However, if there is a lesion on the inner side of the femoral neck, a major trochanteric osteotomy is required. After lesion removal, the major trochanter needs to be fixed with screws, which involves a larger trauma and may require secondary surgery for removal of the internal fixation.

The DAA isfirst described by German surgeon Carl Hueter in the nineteenth century and published in Der Grundriss der Chirurgie (The Compendium of Surgery). This surgical approach is also known as the "Hueter approach" [4]. It was not until 1917, after a report by Smith-Peterson, that the surgical approach became widely known. In 1950, French doctor Judet also reported hip replacement by anterior approach [19], but with the emergence of new artificial joints, this approach gradually decreases and was only occasionally used in the treatment of hip infection in children [4]. In 1980, Light and Keggi reported the experience of 104 patients of modern total hip arthroplasty using the anterior approach. This surgical approach has the advantages of the short operation time, less bleeding, no intraoperative complications, short hospitalization time, fast functional recovery, etc., which has aroused the attention of the medical community again. Become one of the surgical approaches for Total Hip Arthroplasty (THA) [20]. But what really brought it to the forefront of clinicians' discussions was the popularity of minimally invasive surgery in recent decades.

Compared with Simth-Peterson and Watson-Jones, the DAA is not familiar to pediatric surgeons and has been rarely reported in the field of pediatric orthopedics. In fact, this approach has the advantages of less trauma, easy exposure and shorter operation time, which is more suitable for children. In addition, the DAA also relatively "protects" the vessels where the base of the femoral neck is located, which can effectively avoid vascular damage [21, 22]. If the lesion removal in this area causes excessive trauma and disrupts the blood supply, it may increase the risk of complications such as avascular necrosis of the femoral head. Therefore, minimally invasive surgery is the key to avoiding this serious complication. In contrast, the Watson-Jones or Simith-Peterson approaches often result in large surgical trauma and a relatively higher risk of complications. Surgical dislocation approaches involve even greater trauma and may require secondary surgeries.

The DAA involves dissecting through the superficial interval between the sartorius muscle and the tensor fasciae latae, as well as the deep interval between the rectus femoris and gluteus medius muscles. This approach allows for good exposure of the hip joint while avoiding the “extensive dissection” required by other approaches (15). Thus, there is minimal bleeding during hip joint exposure, with an average blood loss of only 23.9 mL in our case series. In addition, the DAA can expose the femoral neck and femoral head without cutting any muscles and tendons and is a scheme to expose the surgical field solely through the space between muscles, basically without damaging or affecting the muscles of the lower extremities. In rare patients, during the final articulation of the hip joint, it is necessary to cut off part of the inverted head of the rectus femoris in order to facilitate the articulation of the hip joint (in most patients, it is not necessary to cut off). The anatomical advantages of this surgical approach allow for rapid recovery of muscle and joint function after surgery. In our case series,unless there were a concern about the fracture, it would be rare for patients to need to remain in bed for rest. Because this approach can directly expose the hip joint, the exposed incision is small and there is no muscle disconnection, so the incision suture is fast. The average suture time in this group is 40.3 min, which relatively shortens the entire operation time. However, the amount of bleeding can vary significantly among different diseases, and therefore the bleeding volume between patients may not be directly comparable. For example, aneurysmal bone cyst bleeding is more, and Kapo's sarcoma bleeding is also a lot, but simple bone cyst bleeding will be less. Therefore, the characteristics of different diseases need to be taken into account when assessing and comparing the amount of blood loss.

This retrospective study has limitations due to its limited sample size. Specifically, the low incidence of femoral head ischaemic necrosis does not completely demonstrate the benefits of the access route, which requires verification in a larger study. Additionally, alternative approaches to removing femoral neck lesions exist, but we have focused solely on the method used in this study.

## Conclusion

The DDA approach has a broad application in the treatment of childhood diseases. It is favored for its advantages of less trauma, quick recovery, short hospital stay, short operation time, and low complications, following the patient to start moving early.

## Data Availability

All data in the study were obtained in good faith and the datasets used and analysed during the current study are available from the corresponding author upon reasonable request.
